# Use of Non-Steroidal Anti-Inflammatory Drugs and Attitudes to Pain in Pasture-Based Dairy Cows: A Comparative Study of Farmers and Veterinarians

**DOI:** 10.3389/fvets.2022.912564

**Published:** 2022-05-30

**Authors:** Natasha Browne, Muireann Conneely, Chris Hudson

**Affiliations:** ^1^Teagasc, Animal & Grassland Research and Innovation Centre, Moorepark, Fermoy, Ireland; ^2^School of Veterinary Medicine and Science, University of Nottingham, Loughborough, United Kingdom

**Keywords:** pain, dairy cow, veterinarian, NSAIDs, analgesia, farmer

## Abstract

Pain is a significant welfare concern within the dairy industry. Recognizing and managing pain are important factors for safeguarding animal welfare. A questionnaire was sent via post to Irish dairy farmers and large animal veterinarians to assess attitudes to pain and the use of non-steroidal anti-inflammatory drugs (NSAIDs) in pasture-based dairy cows. The questionnaire could also be completed online. A total of 1,002 surveys were received from dairy farmers and 116 from livestock veterinarians. Veterinarians and farmers generally perceived the same conditions and procedures as the most painful. However, farmers scored surgical procedures significantly higher than veterinarians, and veterinarians scored lameness-related conditions, mastitis (clots in milk only) and hock hair loss significantly higher than farmers. Higher pain scores for conditions and procedures given by dairy farmers and veterinarians were associated with increased NSAID use. However, the use of NSAIDs was low, relative to the pain score, for Burdizzo castration (farmers and veterinarians), white line separation (farmers and veterinarians) and abscess (veterinarians), mastitis with clots in milk only (farmers) and calving with no assistance (farmers). Veterinarians who graduated less recently had significantly lower odds of using NSAIDs, and farmers that completed the survey online, had a larger herd size, completed education up to level four or five (as opposed to level three) and those who seemed to have less knowledge on analgesics, had significantly lower odds of using NSAIDs. Empathy was not found to be associated with NSAID use and no correlation was found between pain and empathy scores. Veterinarians perceived cost as more of a barrier than farmers did; therefore, NSAIDs should be offered more readily. For those working with dairy cows, there is a need to continue education on the benefits of analgesia, especially for conditions and procedures that have low NSAID use relative to pain score. The habituation of humans to pain in animals needs to be prevented to enable pain to be recognized and managed appropriately. Pain scores can be used as a benchmark for veterinarians and farmers to determine how their perception of pain compares to others, and see how this may influence their NSAID use.

## Introduction

Pain is a sensory and emotional experience which can have major impacts on dairy cow welfare. Under current EU legislation, farm animals are considered sentient beings (1), and are therefore recognized to suffer and feel pain. Freedom from pain is a key component of welfare (2); therefore, preventing and alleviating pain plays a vital role in safeguarding the welfare of cows and calves within the dairy industry. Pain in dairy cows can be caused by various diseases, injuries, parturition, and surgical and non-surgical procedures (3), as well as from routine management practices of calves such as disbudding and castration (4, 5). Pain is also a problem in terms of consumer perception and the supply chain; there is heightened pressure on the agricultural industry to produce food more ethically and sustainably (6, 7).

Pain must be recognized appropriately in order for it to be managed effectively. It is therefore important to understand what qualities and views of individuals lead to the recognition of pain in dairy cows. Although it is the shared responsibility of farmers and prescribing veterinarians to ensure pain is appropriately assessed and treated, the majority of studies focus only on veterinarians (8–11). There is also limited comparative work between the attitudes to pain between both veterinarians and farmers. Tschoner et al. (12) reported that there was no significant differences between pain scores of Bavarian veterinarians and farmers. In contrast, Thomsen et al. (13) reported that Danish dairy farmers gave higher pain scores compared to veterinarians. However, farmers in this study were part of a web-based panel and may therefore not be representative of the full population. Additionally, the farmer survey was undertaken over 2 years following the survey with the veterinarians. Further work is required to understand how attitudes to pain compare between veterinarians and farmers.

Non-steroidal anti-inflammatory drugs (NSAIDs) can be used to treat painful conditions such as mastitis (14), and to relieve post-operative pain in both cows and calves. Non-steroidal anti-inflammatory drugs are the only family of analgesics available for food producing animals in the European Union that provide long acting pain relief [24–72 h of pain relief per dose (15)]. In addition to improving the welfare of the dairy cow through reduced pain, NSAIDs can also accelerate recovery of lame cows (16) and improve productivity (17). Although the use of analgesia is generally increasing within the dairy sector, uptake was found to be low in the treatment of lameness within an Irish pasture-based dairy system (unpublished data), and other studies (8) have found evidence of scope to increase use further. The use of NSAIDs for different cattle procedures and conditions is generally higher if perceived more painful by a veterinarian (8, 9, 11). Using a multilevel model, Remnant et al. (8) also reported that NSAID use was higher in female veterinarians and for those that graduated in more recent decades, after accounting for the effect of pain score. However, to the authors' knowledge, no research to date has used multivariable statistics to determine factors that account for the use of NSAIDs by dairy farmers.

In Ireland, NSAIDs are classified as “prescription only” medicines; however, farmers can administer NSAIDs themselves to both cows and calves in line with the prescription obtained (18). Although the farm's veterinarian does not necessarily have to visit the farm in order to provide a prescription, they should have visited the farm within the last 12 months (19). The veterinarian can prescribe NSAIDs to both individual animals and to a group of animals (19). Based on a veterinary prescription, a small quantity of NSAIDs can also be kept on farms for future use, if deemed necessary by the veterinarian (20). In addition, as of June 2022 it will become mandatory for veterinarians to prescribe using the National Veterinary Prescribing System (NVPS) as opposed to using paper prescriptions, in line with new EU regulations to improve medicine availability and reduce the use of anti-microbials (21). This highlights that both the farmer and veterinarian have control and influence over NSAID use in dairy cows.

Empathy is a personality trait of clinical interest, particularly in the topic of pain recognition and management. However, no studies to date have researched empathy as a factor that may specifically affect NSAID use in dairy cows by either veterinarians or farmers. Empathy is measurable using assessments such as the Interpersonal Reactivity Index [IRI (22)]. Previous studies have found that empathy toward animals decreases with years of study in veterinary students, with female students maintaining higher empathy levels throughout the course of study compared to male students (23). Norring et al. (24) also reported that empathetic veterinarians scored the pain of various conditions and procedures higher than those with lower empathy scores.

The aim of this study was to assess attitudes to pain and use of analgesics in both dairy farmers and livestock veterinarians. A further aim was to identify factors associated with NSAID use in pasture-based dairy cows. The final aim was to establish the relationship between the pain score given to certain procedures and conditions and both NSAID use and empathy scores of farmers and veterinarians.

## Materials and Methods

The study took place from September 2021 to November 2021. Data were collected via two questionnaires, one directed at farmers and one directed at veterinarians in Ireland. Both questionnaires can be viewed as Supplementary Material. To allow for comparisons between studies, parts of each questionnaire were similar to those used previously in the UK (8, 9, 25), Europe (11, 13) and New Zealand (10). The study was approved by the School of Veterinary Medicine and Science Committee for Animal Research and Ethics (CARE) at the University of Nottingham (reference number: 3417 210812).

### Farmer and Veterinarian Selection

Addresses of all veterinary practices registered with the Veterinary Council of Ireland (VCI) in July 2021 (768 practices) were retrieved from the VCI website (https://www.vci.ie). Each practice was checked to determine if the practice profile included large animal services, according to the VCI. For practices that did not have their profile accessible via the VCI, the veterinary practice's own website was checked. A total of 455 practices included large animal services. Each practice owner was sent the paper questionnaire via post, along with a cover letter asking the owner to distribute the questionnaire to veterinarians within the practice. Three surveys were returned undelivered by the postal service; therefore, a final subset of 452 surveys were received by veterinary practices.

A list of addresses were provided by the Irish Cattle Breeding Federation (ICBF) for farmers that had consented to allowing Teagasc access to their information in July 2021 (10,325 farmers). Due to financial and time constraints preventing us sending the survey to all farmers, a subset of 6,500 farmers were randomly selected and sent the paper questionnaire by post. Alongside each questionnaire and cover letter, a prepaid envelope to return the questionnaire were included. The cover letter stated the study aims, and that the study was anonymous, entirely voluntary, for research purposes and that results may be used in publications and conference presentations. Prior to survey distribution, both surveys were reviewed by three researchers outside of the research team. The farmer survey was also piloted on two dairy farmers and the veterinary survey on two veterinarians prior to sending out the survey. Postal surveys were sent to both veterinarians and farmers in September 2021.

An online version of each questionnaire was also produced on Microsoft Forms (Microsoft Corporation, Washington). The link to the corresponding questionnaire was also provided on the paper version, allowing the respondent the option to complete the questionnaire in his or her desired format. This also allowed for multiple veterinarians in a single practice to complete the questionnaire. The links were also posted on social media outputs to advertise the questionnaire to both veterinarians and farmers. Additionally, Veterinary Ireland (representative body for veterinary surgeons in Ireland) publicized the online veterinary survey via email to their members. The online veterinary survey was also advertised to delegates at the Cattle Association of Veterinary Ireland (CAVI) conference. The online surveys were closed and any remaining postal surveys were disregarded after 30th November 2021.

### Questionnaire

The first section of the veterinarian questionnaire obtained demographic information including gender, date of birth, background prior to veterinary school (rural, urban, or a combination), location of veterinary school, year of graduation, postgraduate education, veterinary practice location, practice position and proportion of time spent treating cattle. The second section consisted of nine statements relating to the use of analgesics in dairy cows; veterinarians were asked if they agreed or disagreed with each statement. The third section asked for which procedures and conditions the respondent would provide NSAIDs and for what proportion of cases, and what they would consider an acceptable total cost for a course of pain relief for each procedure and condition. The fourth section asked veterinarians to rate the pain of 12 conditions and nine procedures that relate to cows or calves, when provided no pain relief, on a ten-point scale from one (no pain) to ten (worst imaginable pain). The fifth section consisted of questions to determine the veterinarians' empathy toward animals. As created by Norring et al. (24), this section included statements from the perspective taking (PT) and empathy concern (EC) subscale of the IRI (22), reworded to focus on empathy toward animals rather than humans. The PT subscale rates the respondent's ability to adopt the point of view of others, whereas the EC subscale rates the respondent's ability to feel sympathy and concern for others [IRI (22)]. Veterinarians were asked to score 14 statements on a five-point scale from zero (does not describe me well) to four (describes me very well). The final section related to lameness in dairy cows; questions pertained to the veterinarian's involvement with lameness on their clients' farms, education on lameness and pain management, views on current lameness management on dairy farms and lameness treatment (results from this section are not included in this paper).

The farmer questionnaire was similar to the veterinarian questionnaire described above. The first section obtained demographic information including gender, date of birth, highest level of education (26), background prior to farming, number of years full time farming, farm location and herd size. The second section (opinions on the use of analgesics) was identical to the veterinary survey, as was section four (pain assessment) and five (empathy assessment). The third section asked for which procedures and conditions the respondent would like a cow or calf under their care to receive pain relief that lasted ≥24 h, and what they would consider an acceptable total cost for a course of pain relief for each procedure and condition. The final section included questions relating to lameness in dairy cows, including the use of pain relief, veterinarian involvement and lameness management (results from this section are not included in this paper).

### Statistical Analysis

Data from both the veterinarian and farmer paper questionnaires were input into Excel 2016 (Microsoft Corporation, Washington) and merged with data from the online version of the questionnaire. Data cleaning was undertaken to identify and correct errors within the dataset. All descriptive analysis and modeling was completed using R version 3.3.1 (R Core Team, Vienna, Austria).

Logistic regression models were used to assess the difference in agreement between veterinarians and farmers for the eight statements regarding opinions on analgesics. Statement agreement was the binary outcome variable (1 = respondent agrees; 0 = respondent disagrees). The model predictor was respondent group (veterinarian or farmer). For each statement, an additional logistic regression model was also built through backwards selection using a range of additional predictors (gender, age, background, farm or practice location, region, and empathy score). Predictors were removed one at a time (based on highest *P*-value) until all variables in the model had at least one significant category *(P* <0.05*)*. Multicollinearity was checked using variance inflation factor (27) and goodness of fit using the Hosmer-Lemeshow test (28).

Pain scores for each condition and procedure were compared between veterinarians and farmers using the Mann Whitney U test (Wilcoxon Rank Sum Test), and violin plots were produced to show data distribution. Using Spearman's rank correlation coefficient, the relationship between the percentage of farmers that would like NSAIDs used for each condition and procedure, and the median farmer pain scores was determined. The relationship between the percentage of veterinarians that use NSAIDs in ≥50% of cases for each condition and procedure [same threshold as used by Remnant et al. (8)], and the median veterinarian pain scores was also established. Separately for farmer and veterinarian respondents, the relationship between median pain scores across all conditions and procedures and empathy score at respondent level were also assessed using Spearman's rank correlation coefficient.

Mixed effects logistic regression was performed to model the effects of various predictors on the odds of a farmer wanting NSAIDs to be used. A second mixed effect logistic regression model was performed to model the effects of various predictors on the odds of whether veterinarians used NSAIDs in ≥50% cases. For both models, predictors included the condition and procedure, pain score for each condition and procedure, demographic information and statements regarding analgesia. Predictors were checked for non-zero variance, and were not included in the models if non-zero variance occurred. Data for modeling was structured such that each unit of data represented one procedure or condition for one respondent. A random effect term to reflect respondent was also included. The final models were selected via backwards selection based on significance; variables were kept in the model if at least one category had a *P*-value < 0.05. Multicollinearity was assessed using variance inflation factor (27) and model fit was checked using the Hosmer-Lemeshow test (28). Odds ratios were calculated based on coefficient estimates.

## Results

In total, 1,002 farmer surveys were received. Nine hundred and twenty five were completed due to the farmer receiving the survey via post (of these, 822 returned the paper version and 103 completed the online version instead), resulting in a response rate of 14% percent (925/6,500). The remaining 77 online survey responses were as a result of social media engagement; therefore, a response rate could not be calculated.

A total of 116 veterinarian surveys were received. One-hundred and two surveys were completed due to the practice receiving the postal survey (of these, 86 returned the paper version and 16 completed the survey online). This provided a response rate of 23% (102/452); however, multiple responses may have been from a single veterinary practice which would therefore lower this response rate. An additional 14 surveys were completed online due the survey being advertised via Veterinary Ireland, the CAVI conference and social media. Information in regards to the demographics of both farmer and veterinarian respondents can be found in Table 1.

**Table 1:** Demographics of farmers (*n* = 1,002) and veterinarians (*n* = 116) that completed a survey on attitudes to pain and analgesic use in pasture-based dairy cows.

**Demographics**	**Farmer**	**Veterinarian**
**Age (yrs)**		
Median (IQR)	51 41–59	48 35–59
**Gender (%)**		
Female	5.0	21.7
Male	94.8	78.3
Other	0.2	0.0
**Background (%)**		
Rural	95.2	77.2
Rural & Urban	4.2	14.0
Urban	0.6	8.8
**Veterinary school location (%)**		
Ireland	n/a	82.6
Other	n/a	17.4
**Graduation (yr)**		
Median (IQR)	n/a	1994 (1983–2007)
**Additional qualifications (%)**		
None	n/a	60.2
Certificate	n/a	26.5
Diploma	n/a	5.3
Postgraduate	n/a	9.7
**Highest level education (%)**		
None	1.4	n/a
Level 3 (junior certificate)	22.8	n/a
Level 4 & 5 (leaving certificate)	44.7	n/a
Level 6 (higher/advanced certificate)	2.0	n/a
Level 7 & 8 (bachelor degree)	24.8	n/a
Level 9 & 10 (masters & doctorate)	4.4	n/a
**Farm/veterinary practice location (%)**		
Munster	57.9	46.3
Ulster	6.9	4.6
Leinster	30.5	25.5
Connacht	4.7	23.6
**Position**		
Partner/clinical lead	n/a	70.8
Employee	n/a	29.2
**Proportion time treating cattle (%)**		
Median (IQR)	n/a	65 (50–90)
**Full time farming (yrs)**		
Median (IQR)	31 (20–40)	n/a
**Herd size (cows)**		
Median (IQR)	110 (75–165)	n/a

*For categorical variables the percentage of respondents in each category are reported and for continuous variables the median and interquartile range (IQR) are reported*.

### Opinions on Pain and Analgesics

Differences in the opinions on pain and analgesics between veterinarians and farmers can be viewed in Table 2. Significant differences between farmer and veterinarian agreement were found for four out of the seven statements. In addition to the difference found between veterinarians and farmers, it also appeared that the odds of a farmer and veterinarian agreeing with “Analgesics may mask deterioration in the animal's condition” was higher when the respondent's age was between 40 and 50, or >50, compared to <40, and when the respondent's empathy score was >40 compared to <30. For two of the statements where no difference was found between veterinarians and farmers, other factors were shown to affect agreement. The odds of a farmer and veterinarian agreeing with “Farmers do not know enough about controlling pain in cattle” was lower when the respondent's empathy score was between 31 and 40, and >40, compared to <30. The odds of a farmer and veterinarian agreeing with “Vets do not discuss controlling pain in cattle with farmers enough” was also lower when the respondent's age was between 40 and 50 compared to <40, and when the respondent's empathy score was >40 compared to <30.

**Table 2:** The agreement of farmers and veterinarians with statements relating to analgesia and pain in cattle, in a survey investigating attitudes to pain and analgesic use in pasture-based dairy cows.

**Statement**	**Veterinarian agreement (%)**	**Farmer agreement (%)**	***P*-value**	
Analgesics may mask deterioration in the animal's condition	25	38	0.006	**
Cattle benefit from receiving analgesic drugs as part of their treatment	98	90	0.012	*
Some pain is necessary to stop the animal becoming too active	15	18	0.371	
Cattle recover faster if given analgesic drugs	97	75	0.000	***
Drug side effects limit the usefulness of giving analgesics to cattle	10	12	0.397	
Farmers are happy to pay the costs involved with giving analgesics to cattle	63	75	0.006	**
Farmers would like cattle to receive analgesia but cost is a major issue	26	30	0.356	
Farmers do not know enough about controlling pain in cattle	70	63	0.133	
Vets do not discuss controlling pain in cattle with farmers enough	57	56	0.994	

*Statistical differences between the agreement of veterinarians and farmers are reported for each statement, based on logistic regression models (*

^***^P < 0.001,

^**^P < 0.01,

^*^P < 0.05).

### NSAID Use and Cost

Table 3 reports the proportion of farmers that would like a cow to receive NSAIDs for a range of conditions and procedures, and the proportion of veterinarians that give NSAIDs for ≥50% of cases for a range of conditions and procedures. Surgical procedures, of both calves and cows, were the conditions or procedures for which the highest proportion of farmers (86–98%) stated that they would like NSAIDs used. For surgical procedures, the proportion of veterinarians that would give NSAIDs for ≥50% of cases ranged from 65 to 88%. The proportion of farmers that stated they would like NSAIDs used was generally higher than the proportion of veterinarians that stated they give NSAIDs for ≥50% of cases for all conditions, with the exception of mastitis.

**Table 3:** Proportion of farmers that would like a cow to receive NSAIDs for different conditions and procedures, and the proportion of veterinarians that give NSAIDs for ≥50% of cases for each condition and procedure, in a survey investigating attitudes to pain and analgesic use in pasture-based dairy cows.

**Condition/procedure**	**Proportion farmers that would like a cow to receive NSAIDs for each condition/procedure (%)**	**Proportion veterinarians using NSAIDs for ≥50% of cases for each procedure/condition (%)**										
			**Proportion of respondents who selected the following as an acceptable**									
			**cost for a course of analgesia for each procedure/condition (%)** ^**a**^									
			**Farmers**					**Veterinarians**				
			€**0**	€**0–5**	€**5–15**	€**15–30**	**>**€**30**	€**0**	€**0–5**	€**5–15**	€**15–30**	**>**€**30**
Disbudding (calf)	64	62	3	65	22	8	2	0	79	17	4	0
Burdizzo Castration (calf)	44	32	2	57	27	11	3	0	82	18	0	0
Surgical castration (calf)	86	65	2	42	36	14	7	0	67	29	4	0
Digit amputation	97	88	0	7	26	32	34	0	3	27	43	27
Sole ulcer treatment	80	75	0	21	46	26	7	0	10	45	41	3
Sole hemorrhage	63	54	1	24	47	23	6	0	16	52	31	2
White line abscess	74	64	0	23	46	23	8	0	13	46	34	7
White line (no abscess)	35	34	2	23	46	21	9	0	10	58	30	3
LDA surgery	91	72	1	11	30	26	32	0	9	46	39	6
Mastitis	26	42	1	27	38	23	10	0	21	37	38	4
Cesarean section	98	76	0	6	22	27	45	0	4	44	36	16
Dystocia	73	68	0	15	33	27	24	0	9	51	32	7
Calving (no assistance)	6	n/a	10	20	37	18	14	n/a	n/a	n/a	n/a	n/a

*The acceptable cost selected for a course of analgesia for each condition and procedure is also reported*.

*^a^Includes farmers that stated they would like a cow to receive NSAIDs for each condition/procedure and veterinarians that used NSAIDs for at least one case for each condition/procedure*.

The acceptable cost of a course of analgesia for each condition and procedure selected by both respondent groups is also reported in Table 3. For both veterinarians and farmers, calf procedures had the lowest acceptable cost for a course of analgesia; the highest proportion of respondents selected €0–5. Whereas, cow surgical procedures generally had the highest acceptable cost for both famers and veterinarians. For eight conditions and procedures, a proportion of farmers selected €0 as the acceptable cost, even though they stated they would like NSAIDs used for these conditions and procedures.

### Pain Scores

**Figure 1:**
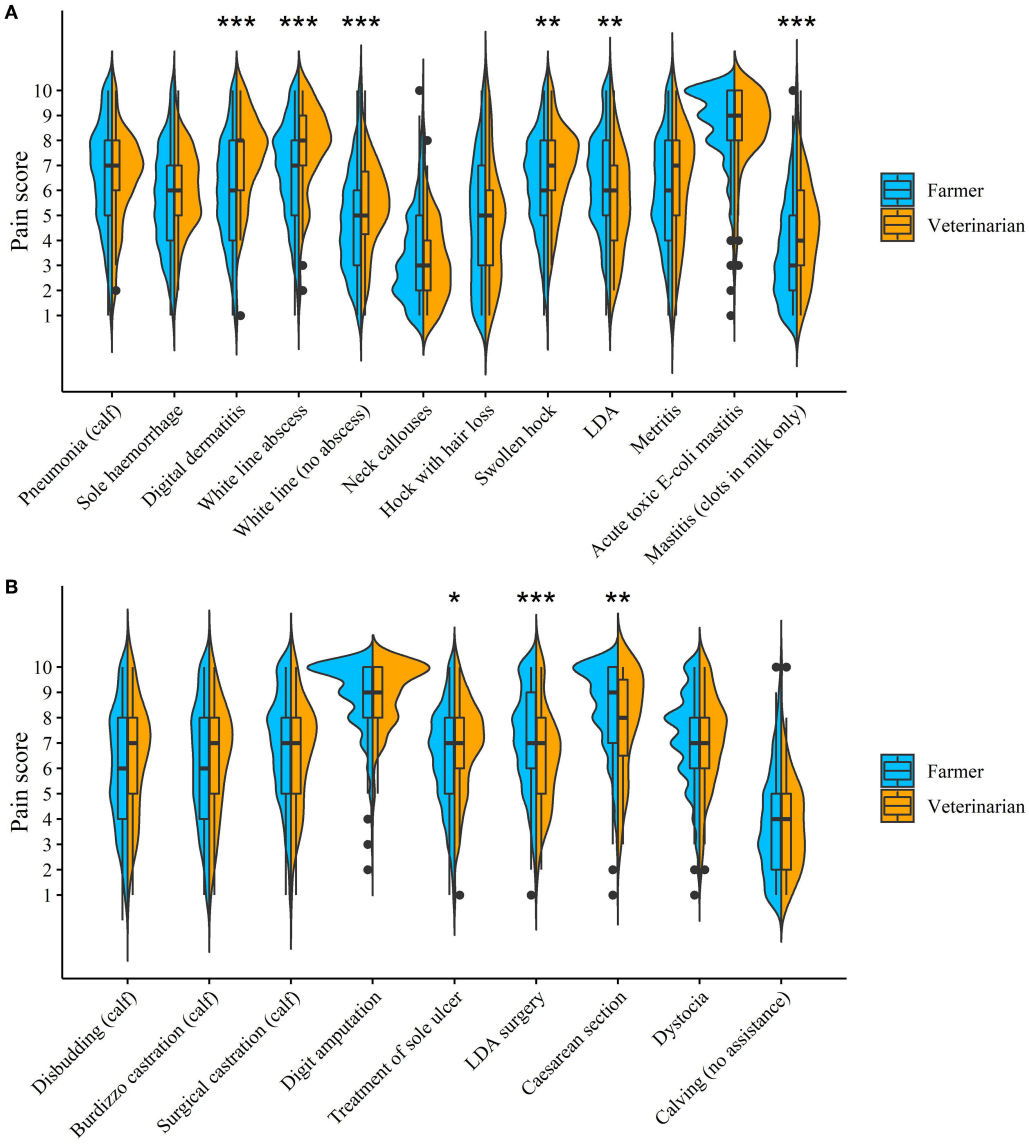
Violin plots showing the distribution of pain scores for different conditions **(A)** and procedures **(B)** split for farmers and veterinarians, in a survey investigating attitudes to pain and analgesic use in pasture-based dairy cows. Overlaid boxplots show the median and interquartile range. Significant differences in pain scores between veterinarians and farmers are indicated (***P < 0.001, **P < 0.01, *P < 0.05).

Figure 1 shows distributions of pain scores, as given by farmers and veterinarians for each condition and procedure, using violin plots. The median of the mean pain score across all conditions and procedures was 6.2 (IQR = 5.4–6.9) for farmers, and 6.4 (IQR = 5.7–7.0) for veterinarians. Both veterinarians and farmers gave the highest median pain score to acute toxic *E-coli* mastitis and digit amputation, giving these a score of nine. Farmers also gave a cesarean section a median pain score of nine. Neither respondent groups had a median pain score of ten for any condition and procedure. The lowest median pain score was three, which was given for neck callouses by both veterinarians and farmers and for mastitis (clots in milk only) by farmers only. Veterinarians scored a swollen hock, mastitis (clots in milk only), digital dermatitis, white line separation (no abscess), white line abscess, and treatment of a sole ulcer significantly higher than farmers. Farmers scored a left displaced abomasum (LDA), LDA surgery and a cesarean section significantly higher than veterinarians.

### NSAID Use and Pain Scores

**Figure 2:**
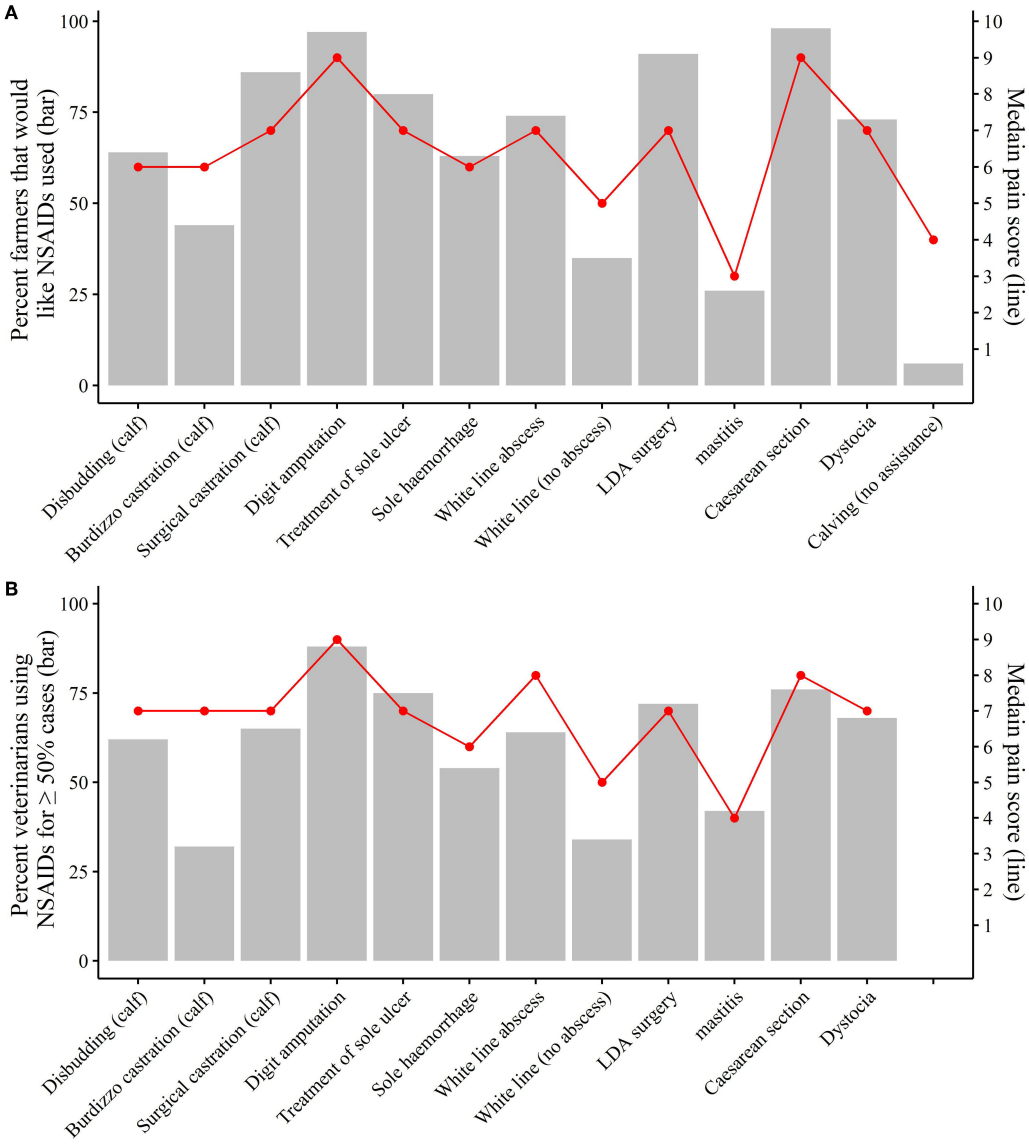
Percentage of farmers that would like NSAIDs used for each condition and procedure (**A**; gray bars) and the percentage of veterinarians that use NSAIDs in ≥50% of cases for each condition and procedure (**B**; gray bars), in a survey investigating attitudes to pain and analgesic use in pasture-based dairy cows. Median pain scores are also shown across each condition and procedure for farmer (**A**; red line) and veterinarians (**B**; red line).

Figure 2 shows the percentage of farmers that would like NSAIDs used for each condition and procedure, plotted alongside median farmer pain scores for the same condition and procedure. This figure also shows the percentage of veterinarians that use NSAIDs in ≥50% of cases for each condition and procedure, alongside median veterinarian pain scores for the same condition and procedure. There was a correlation of 0.9 (*P* < 0.05) between the percentage of farmers that would like NSAIDs used and median farmer pain scores, and a correlation of 0.7 (*P* < 0.05) between the percentage of veterinarians that use NSAIDs in ≥50% of cases and median veterinarian pain scores.

### Factors Associated With NSAID Use

Factors associated with NSAID use by veterinarians and farmers are shown in Tables 4, 5, respectively. Conditions or procedures with pain scores >3 had higher odds of NSAID use by both veterinarians and farmers compared to conditions and procedures with pain scores ≤3. Different conditions and procedures were also associated with different levels of NSAID use by both veterinarians and farmers even after accounting for confounding factors such as the pain score for each of these conditions and procedures. Notable conditions and procedures that resulted in low NSAID use by veterinarians given the relatively high pain score included white line abscess, white line separation (no abscess) and castration of calves using Burdizzo. Conditions and procedures that resulted in low NSAID use by farmers given the relatively high pain score included white line separation (no abscess), mastitis (clots in milk only), calving with no assistance required and castration of calves using Burdizzo.

**Table 4:** Results of a mixed effects logistic regression model that determined factors associated with NSAID use in dairy cows by veterinarians, in a survey investigating attitudes to pain and analgesic use in pasture-based dairy cows.

**Predictor**		**Estimate**	**Odds ratio**	***P*-value**	
**Condition**
	Dystocia	Reference			
	Cesarean	0.642	1.90 (1.90–5.21)	0.212	
	Treatment of sole ulcer	0.455	1.58 (1.58–3.84)	0.316	
	Sole hemorrhage	−0.681	0.51 (0.51–1.18)	0.113	
	White line abscess	−0.891	0.41 (0.41–0.99)	0.045	*
	White line (no abscess)	−1.932	0.14 (0.14–0.35)	0.000	***
	Digit amputation	2.105	8.21 (8.21–34.3)	0.004	**
	LDA surgery	1.318	3.73 (3.73–9.46)	0.005	**
	Mastitis	−0.664	0.51 (0.51–1.25)	0.139	
	Disbudding (calf)	−0.145	0.86 (0.86–2.04)	0.739	
	Surgical castration (calf)	0.124	1.13 (1.13–2.75)	0.784	
	Burdizzo castration (calf)	−2.403	0.09 (0.09–0.23)	0.000	***
**Pain score**
	≤ 3	Reference			
	4	1.119	3.06 (3.06–7.10)	0.009	**
	5	1.571	4.81 (4.81–10.52)	0.000	***
	6	2.028	7.6 (7.60–16.78)	0.000	***
	7	2.284	9.81 (9.81–21.55)	0.000	***
	8	3.127	22.80 (22.80–52.83)	0.000	***
	9	4.267	71.29 (71.29–227.47)	0.000	***
	10	3.896	49.18 (49.18–160.94)	0.000	***
**Graduation year**
	<1991	Reference			
	1991–2005	1.902	6.70 (6.70–16.8)	0.000	***
	2006–2021	2.595	13.40 (13.40–35.7)	0.000	***
**Statement F** ^**a**^
	Disagree	Reference			
	Agree	0.843	2.32 (2.32–5.10)	0.035	*
**Statement H** ^**b**^
	Disagree	Reference			
	Agree	0.961	2.61 (2.61–6.12)	0.027	*

*The binary outcome variable was whether the veterinarian used NSAIDs in ≥50% cases for each procedure and condition (1 = respondent used NSAIDs in more ≥50% cases, 0 = respondent did not use NSAIDs in more ≥50% cases)*.

^***^, ^**^, ^*^ odds ratio is significantly different from 1 (P <0.001, 0.01, 0.05)

*^a^Farmers are happy to pay the costs involved with giving analgesics to cattle*

*^b^Farmers do not know enough about controlling pain in cattle*.

**Table 5:** Mixed effects logistic regression model to determine factors associated with the use of NSAIDs in dairy cows by farmers, in a survey investigating attitudes to pain and analgesic use in pasture-based dairy cows.

		**Estimate**	**Odds Ratio**	***P*-value**	
**Condition**
	Dystocia	Reference			
	Cesarean	2.977	19.64 (11.48–33.79)	0.000	***
	Treatment of sole ulcer	0.859	2.36 (1.81–3.1)	0.000	***
	Sole hemorrhage	−0.053	0.95 (0.74–1.23)	0.680	
	White line abscess	0.215	1.24 (0.96–1.62)	0.103	
	White line (no abscess)	−1.294	0.27 (0.22–0.36)	0.000	***
	Digit amputation	2.533	12.59 (7.62–20.91)	0.000	***
	LDA surgery	2.153	8.61 (6.24–11.95)	0.000	***
	Mastitis	−1.519	0.22 (0.17–0.29)	0.000	***
	Disbudding (calf)	−0.146	0.86 (0.68–1.12)	0.250	
	Surgical castration (calf)	1.173	3.23 (2.44–4.31)	0.000	***
	Burdizzo castration (calf)	−1.451	0.23 (0.19–0.3)	0.000	***
	Calving (no assistance)	−3.940	0.02 (0.02–0.03)	0.000	***
**Pain score**
	≤ 3	Reference			
	4	0.711	2.04 (1.64–2.56)	0.000	***
	5	1.346	3.84 (3.10–4.81)	0.000	***
	6	1.581	4.86 (3.90–6.12)	0.000	***
	7	2.013	7.48 (5.99–9.49)	0.000	***
	8	2.506	12.26 (9.59–15.80)	0.000	***
	9	3.098	22.14 (16.12–30.88)	0.000	***
	10	3.170	23.81 (16.78–34.13)	0.000	***
**Survey format**
	Online	Reference			
	Paper	0.524	1.69 (1.30–2.23)	0.000	***
**Education (highest level)**
	3	Reference			
	4 & 5	−0.429	0.65 (0.51–0.84)	0.001	***
	6	0.228	1.26 (0.56–2.83)	0.579	
	7 & 8	−0.166	0.85 (0.63–1.14)	0.271	
	9 & 10	−0.030	0.97 (0.58–1.65)	0.913	
	None	0.458	1.58 (0.66–3.82)	0.308	
**Herd size (cows)**
	30–100	Reference			
	101–150	−0.292	0.75 (0.59–0.96)	0.018	*
	>150	−0.294	0.75 (0.58–0.96)	0.020	*
**Statement A** ^**a**^
	Disagree	Reference			
	Agree	−0.211	0.81 (0.66–1.00)	0.043	*
**Statement D** ^**b**^
	Disagree	Reference			
	Agree	0.660	1.94 (1.56–2.44)	0.000	***
**Statement H** ^**c**^
	Disagree	Reference			
	Agree	0.257	1.29 (1.05–1.62)	0.022	*
**Statement I** ^**d**^
	Disagree	Reference			
	Agree	0.262	1.3 (1.06–1.62)	0.016	*

*The binary outcome variable was whether farmers would like NSAIDs used for a cow under their care for each procedure and condition (1 = respondent wants NSAIDs used, 0 = respondent does not want NSAID used)*.

^***^, ^*^ odds ratio is significantly different from 1 (P <0.001, 0.05)

*^a^Analgesics may mask deterioration in the animal's condition*

*^b^Cattle recover faster if given analgesic drugs*

*^c^Farmers do not know enough about controlling pain in cattle*

*^d^Vets do not discuss controlling pain in cattle with farmers enough*.

After accounting for condition and pain score, veterinarians that graduated more recently, and veterinarians that agreed that “Farmers are happy to pay the costs involved with giving analgesics to cattle” and “Farmers do not know enough about controlling pain in cattle” had higher odds of NSAID use. After accounting for condition and pain score, farmers who completed the paper survey (as opposed to the online survey), only completed education up to level three (as opposed to level four and five), had a smaller herd size, agreed that “Cattle recover faster if given analgesic drugs”, “Farmers do not know enough about controlling pain in cattle” and “Vets do not discuss controlling pain in cattle with farmers enough,” and disagreed that “Analgesics may mask deterioration in the animal's condition” had higher odds of NSAID use.

### Empathy and Pain Scores

The median farmer empathy score was 38 (IQR = 31–44) and the median for the subscales empathetic concern and perspective taking were 21 (IQR = 18–25) and 17 (IQR = 13–20), respectively. The median veterinarian empathy score was 37 (IQR = 30–45) and the median for the subscales empathetic concern and perspective taking were 20 (IQR = 16–25) and 17 (IQR = 12–21), respectively. No significant correlation was found between median pain scores and empathy scores for either farmers or veterinarians.

## Discussion

Pain compromises animal welfare and can reduce dairy cow productivity. To enable pain to be alleviated, pain must firstly be recognized by both farmers and veterinarians. Farmers and veterinarians gave similar pain scores when averaged across all conditions and procedures. Contrastingly, Thomsen et al. (13) reported that Danish farmers generally scored pain as more severe compared to veterinarians. In the current study, farmers and veterinarians commonly perceived the same conditions and procedures as most painful; acute toxic *E-coli* mastitis and digit amputations were considered to be associated with the most severe pain. Similar findings have been reported previously, whereby digit amputation was reported as most severe by UK (8, 9) and New Zealand (10) veterinarians and E.coli mastitis by Danish veterinarians and farmers (13). Pain scores in this study can be used as a baseline for farmers and veterinarians to determine whether pain is being underestimated by themselves and to further assess whether they are appropriately treating this pain.

Differences in pain scores were, however, found between veterinarians and farmers for some conditions and procedures. Farmers scored LDA, LDA surgery and a cesarean section significantly higher than veterinarians. A possible explanation is that veterinarians see these procedures and conditions as routine, whereas for farmers these are rare and severe occurrences. In contrast, veterinarians gave significantly higher pain scores to lameness related conditions [digital dermatitis, white line separation (no abscess), white line abscess, and treatment of a sole ulcer], mastitis (clots in milk only) and hock hair loss compared to farmers. Becker et al. (29) also reported that the treatment of sole ulcer was scored significantly more painful by veterinarians than farmers. Similarly to the explanation above, it would be uncommon for veterinarians to be called out to farms for these conditions or procedures; they are often mild and treatable by the farmer, or a hoof trimmer may treat lameness related issues. Contrastingly, farmers would see these conditions and procedures on a day-to-day basis. This demonstrates that farmers and veterinarians can become habituated to the pain of certain conditions because of frequent exposure. It is important for cow welfare that efforts are made to prevent this “habituation” of pain.

Once pain is recognized, it can then be treated. Non-steroidal anti-inflammatory drugs block the release of prostaglandins, reducing inflammation, fever and pain (30). As opposed to anesthesia, which is generally used for short-term pain relief during surgical procedures, NSAIDs can provide longer-acting relief from pain. In agreement with previous studies, the higher the pain score for a particular condition or procedure, the more likely a veterinarian was to give NSAIDs (8, 9). Similarly, the recognition of more severe pain by dairy farmers was associated with an increased willingness for NSAIDs to be given. These associations also remained true when other factors, such as gender, were accounted for in multilevel models. This shows that both veterinarians and farmers are reasonably good at recognizing pain, and treat accordingly through the administration of NSAIDs.

Cow surgical procedures generally resulted in the highest NSAID use by both veterinarians and farmers. Despite this, the indicated NSAID use was still lower than that reported for the UK for these surgical procedures (8). It must be noted that these procedures will still be carried out under local anesthetic; however, local anesthesia alone does not offer the long-term post-operative pain relief that NSAIDs can provide. In contrast to cow surgical procedures, the use of NSAIDs by veterinarians for surgical castration of calves and disbudding was much higher in this study compared to the UK (8). However, the UK study was carried out 5 years prior to the current study, and it is expected that NSAID use in calves will have subsequently increased over this timeframe.

Some conditions and procedures seemed to have low NSAID use despite being assigned relatively high pain scores. Remnant et al. (8) also identified that the type of condition or procedure influenced NSAID use by veterinarians; however, no studies to date have used multilevel modeling to assess the willingness for NSAIDs to be given by dairy farmers. The use of NSAIDs in calves, despite the relatively high pain score, was low for castration using Burdizzo for both veterinarians and farmers. Similar was found for veterinarians in the UK; however, Remnant et al. (8) also identified disbudding to have low NSAID use relative to the pain perceived, whereas the current study showed a higher level of NSAID use for disbudding. Studies have shown that providing NSAIDs for calf castration has physiological and behavioral benefits (31, 32). The British Veterinary Association and Veterinary Ireland have both produced a position statement stating that they consider it best practice to provide NSAIDs for castration (33, 34); however, this study shows that more needs to be done to increase awareness on NSAID use and the benefits to calf welfare.

Veterinarians were also significantly less likely to use NSAIDs, after accounting for the effect of pain score, for white line separation (no abscess) and white line abscess, and farmers were significantly less likely to want NSAIDs given for white line separation (no abscess) and mastitis (clots only). Mastitis and lameness are both common endemic diseases within the dairy industry, and while prevention is vital for controlling these diseases, appropriate treatment is equally important. Providing NSAIDs has been reported to reduce clinical signs of mastitis (35–37) and improve production measures (37, 38). In terms of lameness, Thomas et al. (16) reported that cure rates were improved if NSAIDs were provided on top of a therapeutic trim and block, in newly lame cows. However, a pasture-based study in New Zealand disagreed with these findings, showing no difference in locomotion score or nociceptive threshold between treatment groups (39). More studies are required to evaluate the benefits of NSAIDs for lameness in terms of cure rates, pain reduction and impact on production measures in different system types.

The biggest difference in the willingness of farmers to want NSAIDs given, relative to the pain score, was seen with calving (when no assistance was required). Despite a pain score of four, only 6% of farmers wanted NSAIDs given at calving. There is inconsistency in the reported effects of NSAIDs on cow welfare and performance at calving. Some studies have reported positive results of improved milk yield, reproductive performance and a reduction in uterine diseases (40, 41), whereas, other studies have shown no improvement in these factors (42). Additionally, Wilson et al. (43) reported reduced lameness and culling when heifers were given NSAIDs at their first and subsequent calvings; however, no effects where seen in cows that had already calved prior the commencement of the study. Despite the variation in results in terms of physiological benefits, it must also be considered that NSAIDs could improve cow welfare by reducing the pain during parturition (44, 45).

In addition to pain score and condition, year of graduation from veterinary school was also associated with NSAID use by veterinarians. Veterinarians graduating more recently had higher odds of giving NSAIDs, which is consistent with findings from previous studies (8). It is theorized that this may due to views on animal welfare and the perception of pain changing over the generations, or that veterinarian's sensitivity to pain may decrease with experience of treating painful conditions and carrying out procedures. As such, it is important that veterinarians continue professional development though courses and workshops relating to the recognition of pain to ensure pain perception is not desensitized over time.

Farmers that completed the survey via post had significantly higher odds of wanting NSAIDs used than those that completed the survey online. A possible theory is that completing the survey on paper and making the effort to post the survey, may demonstrate more commitment and an interest in the area of pain relief. Farmers that had completed education up to level three used more NSAID than those that completed education up to level four or five. There is no clear explanation as to why education to a higher level would seem to result in lower NSAID use; further research may be required to understand the reasons behind this finding.

Interestingly, herd size was associated with the odds of a farmer wanting NSAIDs used on their dairy cows. Farmers with larger herds had lower odds of NSAID use compared to those with smaller herd sizes. Farmers with smaller herds may be more aware and able to recognize the pain of individual cows. Those with larger herds may also be more mindful of profit margins and want to minimize the cost of NSAID use. Currently the majority of farmers believe that they are not educated enough on controlling pain and that veterinarians do not discuss controlling pain enough with farmers. Similar finding were found in UK farmers, whereby 62% of farmers did not feel educated enough on pain relief and 53% felt that veterinarians did not discuss the use of pain relief enough with farmers (25). This study also showed that knowledge on pain relief increased the willingness of farmers to use NSAIDs. Farmers should be educated on the benefits on NSAID use in terms of both welfare and profitability. A quarter of veterinarians also agreed with the statement “Analgesics may mask the determination in the animal's condition.” This shows that veterinarians may also benefit from further education on pain relief within the dairy sector. This may also lead to improved knowledge transfer from veterinarians to farmers.

Empathy was not shown to affect NSAID use by either veterinarians or farmers in the multilevel model used. However, it appeared that empathy affected agreement with some statements regarding veterinarian and farmer opinions on analgesia and pain, of which some of these statements were shown to affect NSAID use in the multilevel model. Therefore, there does seem to be some link between empathy and NSAID use; however, it is unclear to what extent. The same was true for age of the respondent. There was also no correlation found between pain scores and empathy scores in this study, which contrasts to Norring et al. (24) where more empathetic veterinarians gave higher pain scores to bovine conditions and procedures. Comparing these studies, Irish veterinarians and farmers both showed lower empathy compared to Finnish veterinarians when using the same animal focused IRI (24). The difference in empathy scores may be due to gender differences between the two study populations. The majority of veterinarians in the Finnish study were female (91%), whereas, the majority of veterinarian (78%) and farmers (94%) in the current study were male. Previous work has reported that males are generally less empathetic than females, and therefore score lower on the IRI (46–48). The lower empathy score may also be due to cultural differences between countries.

With production costs rising (49) and tight profit margins, cost is also an important factor to consider when looking at NSAID use. Significantly more farmers than veterinarians agreed that “Farmers are happy to pay the costs involved with giving analgesics to cattle.” This indicates that farmers are more willing to pay for NSAIDs than veterinarians perceive. Going forward, NSAIDs should be offered more readily by veterinarians when treating cattle. Compared to previous studies, the cost of NSAIDs seems to be less of a concern. Remnant et al. (8) reported that 45% of UK veterinarians agreed that “Farmers would like cows to receive analgesia but cost is a major issue,” whereas in the current study only 25% of veterinarians agreed with this statement.

Calf procedures, including castration and disbudding, were given the lowest acceptable cost for a course of analgesia by both veterinarians and farmers. This may indicate that farmers are willing to pay more for pain relief of cows compared to calves. The lower acceptable cost may also be due to the smaller doses of pain relief required due to the reduced body weight of calves. The approximate cost of NSAIDs for disbudding and castration per calf is <€2, however, this price will vary depending on the NSAID brand and supplier, and the body weight of the calf. It must be noted that in Ireland disbudding of calves of up to 15 days, castration using Burdizzo up to 6 months and castration using a rubber ring up to 8 days can be performed by the farmer without the use of anesthetic (50, 51). Thermal cauterization is the only method of disbudding that is legal in Ireland (50, 51). There is also no legal requirements to use NSAIDs for disbudding and castration at any age currently in Ireland, however, it has been recommended (52). Some farmers also selected that €0 was an acceptable cost for a course of NSAIDs for some conditions and treatments, despite saying they wanted NSAIDs used. This indicates that a small number of farmers would like NSAIDs used but at no additional cost. This was particularly true for calving, where 10% of farmers wanted NSAIDs used but believed the acceptable cost to be zero.

It must be acknowledged that farmers and veterinarians had the choice to participate in this survey. Respondents may have chosen to participate due to an interest in pain relief management, equally respondents may have chosen to complete the survey to gain further insight into the use of pain relief in dairy cows. It is therefore hard to state definitely that the study population is fully representative; however, a large sample size was obtained, and as such, is as representative as possible. The response rate in this survey was 23% for veterinarians and 14% for farmers. The veterinary response rate in this study was higher than that in the study by Remnant et al. (8), however, lower than in other pain studies (9, 11, 39). The farmer response rate was similar to that of Huxley and Whay (25) and lower than that of Thomsen et al. (13). As with all voluntary surveys non-response bias must be considered when interpreting the results. Voluntary recruitment may lead to bias in prevalence reported estimates (53), such as pain scores in this study. However, sampling via voluntary surveys compared to mandatory sampling has been shown not to affect associations between variables in human health risk factor studies (53). If the same applies to this study, associations between various factors and NSAID use should not be affected by sampling bias. A high proportion of veterinary respondents in this study were also partners or clinical leads, therefore results may not be representative of all veterinarians in Ireland. Additionally, only 22% of veterinarian respondents were female; however, according to the VCI, 44% of veterinarians in Ireland are female (54). However, this statistic included both large and small animal veterinarians. It is therefore expected that a lower number of females specialize in large animal practice compared to small animal.

## Conclusion

Farmers and veterinarians generally considered the same conditions and procedures as more severe; however, some differences in pain scores were seen for particular conditions and procedures. Lower pain scores were generally given by veterinarians for conditions and procedures which would be seen more regularly by veterinarians compared to farmers, and vice versa, highlighting potential habituation to pain. The recognition of pain was found to be an important attribute for increased NSAID use by both farmers and veterinarians. However, some conditions and procedures were shown to have low NSAID use relative to the pain score given. Pain scores should be used as a benchmark for veterinarians and farmers to determine their perception of pain and how this affects their NSAID use. Cost of analgesia did not seem to be as big a barrier to use for farmers as veterinarians perceived. NSAIDs should therefore be offered more readily prior to painful procedures and where the animal is experiencing a potentially painful condition. Education on the benefits of analgesia is vital for increasing NSAID use within the dairy industry and for improving animal welfare.

## Data Availability Statement

The datasets presented in this article are not readily available because data from this study contains potentially identifiable human information (gender, location, herd size etc.) for individual farmers and veterinarians. Requests to access the datasets should be directed to Natasha Browne, natasha.browne@hotmail.co.uk.

## Ethics Statement

The studies involving human participants were reviewed and approved by the School of Veterinary Medicine and Science Committee for Animal Research and Ethics (CARE) at the University of Nottingham (reference number: 3417 210812). The patients/participants provided their written informed consent to participate in this study.

## Author Contributions

MC received funding for the study. CH and MC supervised this study. NB sent the surveys and input all data. NB analyzed the data and drafted the manuscript and with the assistance of CH and MC. All authors contributed to study design, read, and approved the final manuscript.

## Funding

This research was funded by Dairy Research Ireland and the Teagasc Walsh Scholarship Program.

## Conflict of Interest

The authors declare that the research was conducted in the absence of any commercial or financial relationships that could be construed as a potential conflict of interest.

## Publisher's Note

All claims expressed in this article are solely those of the authors and do not necessarily represent those of their affiliated organizations, or those of the publisher, the editors and the reviewers. Any product that may be evaluated in this article, or claim that may be made by its manufacturer, is not guaranteed or endorsed by the publisher.
