# Supplementary material for: Use of Non-Steroidal Anti-Inflammatory Drugs and Attitudes to Pain in Pasture-Based Dairy Cows: A Comparative Study of Farmers and Veterinarians
Source: Front Vet Sci. 2022 May 30;9:912564. doi: 10.3389/fvets.2022.912564 (PMC9190979; doi:10.3389/fvets.2022.912564)
Supplement: Supplementary file 1 [file Data_Sheet_1.PDF]

Farmer survey on pain relief and lameness in Irish dairy cows

If preferred, the survey can also be completed online using the following link (https://forms.office.com/r/79G0VkHTuH) or by scanning the QR code on the cover letter.

PART 1: DEMOGRAPHICS

Please circle the answer or fill in the grey box as required:

1. **Gender:**
2. **Year you were born:**
3. **Highest level of education:** Junior Certificate Leaving Certificate Bachelor Degree Postgraduate degree (i.e. MSc, PhD)
4. **Background before farming:** Rural Urban Rural & Urban
5. **Number of years farming (full time):**
6. **Farm location (county):**
7. **Herd size:**

PART 2: YOUR OPINION ON THE USE OF ANALGESICS IN DAIRY COWS

For each statement below on the use of analgesics (pain relief), tick the box that reflects your opinion best.

Statement	Agree	Not sure	Disagree
Analgesics may mask deterioration in the animal's condition.	☐	☐	☐
Cattle benefit from receiving analgesic drugs as part of their treatment.	☐	☐	☐
Some pain is necessary to stop the animal becoming too active.	☐	☐	☐
Cattle recover faster if given analgesic drugs.	☐	☐	☐
Drug side effects limit the usefulness of giving analgesics to cattle.	☐	☐	☐
Farmers are happy to pay the costs involved with giving analgesics to cattle.	☐	☐	☐
Farmers would like cattle to receive analgesia but cost is a major issue.	☐	☐	☐
Farmers do not know enough about controlling pain in cattle.	☐	☐	☐
Vets do not discuss controlling pain in cattle with farmers enough.	☐	☐	☐

PART 3: USE OF ANALGESICS

For each procedure and condition below, select whether **you** would like an **adult dairy cow or calf** under your care to receive pain relief that **lasts more than 24 hours**, and what you would consider an acceptable cost for a course of pain relief for this procedure/ condition? Tick **one box per question**, for each procedure/ condition.

**Procedure/ condition**	**Would you like a cow under your care to receive pain relief that lasts ≥ 24 hours**	**What would you consider an ACCEPTABLE TOTAL cost for a course of pain relief for each procedure/ condition?**				
		**€0**	**€0 - €5**	**€5 - €15**	**€15 - €30**	**>€30**
Treatment of a sole ulcer (Cow)	Yes ☐ No ☐	☐	☐	☐	☐	☐
Sole haemorrhage/ bruising (Cow)	Yes ☐ No ☐	☐	☐	☐	☐	☐
White line disease with sub-sole abscess (Cow)	Yes ☐ No ☐	☐	☐	☐	☐	☐
White line disease NO sub-sole abscess (Cow)	Yes ☐ No ☐	☐	☐	☐	☐	☐
Claw amputation (Cow)	Yes ☐ No ☐	☐	☐	☐	☐	☐
Caesarean section (Cow)	Yes ☐ No ☐	☐	☐	☐	☐	☐
Dystocia - foetal-maternal disproportion requiring traction (Cow)*	Yes ☐ No ☐	☐	☐	☐	☐	☐
Calving - no assistance required (Cow)	Yes ☐ No ☐	☐	☐	☐	☐	☐
Left displaced abomasum surgery (Cow)	Yes ☐ No ☐	☐	☐	☐	☐	☐
Mastitis - clots in milk only (cow)	Yes ☐ No ☐	☐	☐	☐	☐	☐
Disbudding (Calf)	Yes ☐ No ☐	☐	☐	☐	☐	☐
Surgical castration (Calf)	Yes ☐ No ☐	☐	☐	☐	☐	☐
Castration with Burdizzo (Calf)	Yes ☐ No ☐	☐	☐	☐	☐	☐

* Difficult calving due to an oversized calf, requiring a moderate “pull”

PART 4: PAIN ASSESSMENT

In your opinion, how painful do you think the following conditions and procedures are for **adult dairy cows and calves**? Assume **NO** pain relief is provided. Circle **ONE** number from 1 (no pain) to 10 (worst pain imaginable).

**Condition**	***No Pain***									***Worst pain***
Left displaced abomasum (Cow)	1	2	3	4	5	6	7	8	9	10
Neck Callouses *e.g*. caused by feed barrier (Cow)	1	2	3	4	5	6	7	8	9	10
Acute metritis (Cow)	1	2	3	4	5	6	7	8	9	10
Swollen hock (Cow)	1	2	3	4	5	6	7	8	9	10
Hock with hair loss (Cow)	1	2	3	4	5	6	7	8	9	10
Acute toxic *E-coli* mastitis (Cow)	1	2	3	4	5	6	7	8	9	10
Mastitis - clots in milk only (Cow)	1	2	3	4	5	6	7	8	9	10
Digital dermatitis (Cow)	1	2	3	4	5	6	7	8	9	10
White line disease with sub-sole abscess (Cow)	1	2	3	4	5	6	7	8	9	10
White line disease NO sub-sole abscess (Cow)	1	2	3	4	5	6	7	8	9	10
Sole haemorrhage/ bruising (Cow)	1	2	3	4	5	6	7	8	9	10
Pneumonia (Calf)	1	2	3	4	5	6	7	8	9	10

**Procedure**	***No Pain***									***Worst pain***
Treatment of a Sole Ulcer (Cow)	1	2	3	4	5	6	7	8	9	10
Claw Amputation (Cow)	1	2	3	4	5	6	7	8	9	10
Caesarean section (Cow)	1	2	3	4	5	6	7	8	9	10
Dystocia - foetal-maternal disproportion requiring traction (Cow) *	1	2	3	4	5	6	7	8	9	10
Calving - no assistance required (Cow)	1	2	3	4	5	6	7	8	9	10
Left Displaced Abomasum surgery (Cow)	1	2	3	4	5	6	7	8	9	10
Disbudding (Calf)	1	2	3	4	5	6	7	8	9	10
Surgical castration (Calf)	1	2	3	4	5	6	7	8	9	10
Castration with Burdizzo (Calf)	1	2	3	4	5	6	7	8	9	10

* Difficult calving due to an oversized calf, requiring a moderate “pull”

PART 5: EMPATHY QUESTIONS

For each statement below, circle **ONE** number from 0 (does not describe me well) to 4 (describes me well).

Statement	***Does NOT describe me well***				***Describes me very well***
I often have tender, concerned feelings for animals less fortunate than others.	0	1	2	3	4
I sometimes find it difficult to see things from the animals point to view.	0	1	2	3	4
Sometimes I don't feel very sorry for animals when they have problems or suffer.	0	1	2	3	4
I try to understand the reasons behind an animal's undesired behaviour before making a decision.	0	1	2	3	4
When I see an animal being treated badly, I feel protective towards it.	0	1	2	3	4
I sometimes try to understand animals better by imagining how things look from their perspective.	0	1	2	3	4
Animals' misfortunes do not usually disturb me a great deal.	0	1	2	3	4
If I'm sure I'm right about how to handle an animal, I don't waste time trying to think what might be causing the animals behaviour.	0	1	2	3	4
When I see animals being treated unfairly, I sometimes don't feel very much pity for them.	0	1	2	3	4
I am often quite touched by things that I see happen.	0	1	2	3	4
I believe that there are two sides to every question and try to look at them both.	0	1	2	3	4
I would describe myself as an animal lover.	0	1	2	3	4
When I am disappointed or angry because of how an animal behaves, I usually try to put myself in its place for a while.	0	1	2	3	4
Before scolding an animal, I try to imagine how I would feel if I were in its place.	0	1	2	3	4

PART 6: LAMENESSS IN ADULT DAIRY COWS

Please circle the answer or fill in the grey box as required:

1. **How many cows in your herd have been lame in the <u>last year?</u>**

   Of these lame cows, how many (if any) received pain relief?

2. **How do you decide whether to give a cow pain relief for lameness or not?**

3. **If you do not use pain relief for lameness, why not (select all that apply)?**

   I do use pain relief   Never occurred to me   Vet never suggested it   Too expensive

   I don't think the cow needs it   Other:

4. **What hoof lesion do you believe is the biggest cause of lameness in your herd?**

5. **Do you feel like you are doing enough to reduce lameness in your herd?** Yes No Unsure

6. **Do you consider your knowledge of lameness management adequate?** Yes No Unsure

7. **Do you feel there are enough resources available to you, to increase your knowledge in lameness management?** Yes No

8. **What has prevented you from doing more to reduce lameness in your herd?**

9. **What would motivate/help you to improve lameness in your herd?**

10. **Is your herd lameness scored?** [*Lameness scoring, also known as mobility scoring, is a method used to detect lame cows. It involves scoring each cow individually as they walk, from 0 (good mobility) to 3 (severely impaired mobility)*]

    Yes – How many times per year?   No

    If you answered 'No', what is your reasoning for not lameness scoring your herd?

    If you answered 'No', do you think having your herd regularly lameness scored would benefit your herd? Yes No Unsure

11. **Who treats your lame cows?** Yourself/farm staff   Trimmer   Vet   None

12. **Do you have a lame cow group that you keep closer to the parlour (i.e. so they do not have to walk as far)?** Yes No

    If you answered 'No', what is your reasoning for not having a separate lame group?

13. **On average how many days is there between detecting a lame cow and getting her treated (in the last year)?**

14. **What is the longest time between detecting a lame cow and getting her treated (in the last year)?**

15. **If a cow is not treated within 24 hours of detecting she is lame, what is the main reason for the delay?**

16. **Have you received any advice on lameness from your vet in the last year?** Yes No

17. **Would you like your vet to be more involved in managing lameness on your farm?** Yes No

18. **Do you have a herd health plan written down, which includes lameness protocols (i.e. methods for detecting lameness, what to do if you detect a lame cow, methods of preventing lameness such as footbathing etc.)?** Yes No

    If you answered 'No', what is your reasoning for not having a herd health plan for lameness?

    If you answered 'No', do you think your farm would benefit from having a herd health plan for lameness? Yes No Unsure

Thank you for giving your time to complete this questionnaire

Please return using the pre-paid envelope
