# Supplementary material for: Use of Non-Steroidal Anti-Inflammatory Drugs and Attitudes to Pain in Pasture-Based Dairy Cows: A Comparative Study of Farmers and Veterinarians
Source: Front Vet Sci. 2022 May 30;9:912564. doi: 10.3389/fvets.2022.912564 (PMC9190979; doi:10.3389/fvets.2022.912564)
Supplement: Supplementary file 2 [file Data_Sheet_2.PDF]

Vet survey on pain relief and lameness in Irish dairy cows

If preferred, the survey can also be completed online using the following link (https://forms.office.com/r/Bm385FZwpb) or by scanning the QR code on the cover letter.

PART 1: DEMOGRAPHICS

Please circle answer or fill in the grey box as required:

1. **Gender:**			
2. **Year you were born:**			
3. **Background before veterinary education:**	Rural	Urban	Rural & Urban
4. **Location of veterinary school:**	Ireland	UK	Other
5. **Year of graduation:**			
6. **Postgraduate education undertaken/ undertaking:**	None	Certificate	Diploma
	Postgraduate degree (MSc, PhD)		
7. **Veterinary practice location (county):**			
8. **Practice position:**	Partner/clinical lead	Employee	
9. **Proportion of time spent treating cattle (%):**			

PART 2: YOUR OPINION ON THE USE OF ANALGESICS IN DAIRY COWS

For each statement below on the use of analgesics, tick the box that reflects your opinion best.

Statement	Agree	Not sure	Disagree
Analgesics may mask deterioration in the animal's condition.	☐	☐	☐
Cattle benefit from receiving analgesic drugs as part of their treatment.	☐	☐	☐
Some pain is necessary to stop the animal becoming too active.	☐	☐	☐
Cattle recover faster if given analgesic drugs.	☐	☐	☐
Drug side effects limit the usefulness of giving analgesics to cattle.	☐	☐	☐
Farmers are happy to pay the costs involved with giving analgesics to cattle.	☐	☐	☐
Farmers would like cattle to receive analgesia but cost is a major issue.	☐	☐	☐
Farmers do not know enough about controlling pain in cattle.	☐	☐	☐
Vets do not discuss controlling pain in cattle with farmers enough.	☐	☐	☐

PART 3: USE OF ANALGESICS

The following question relates to **your** use of **NSAIDs** (e.g. meloxicam, flunixin, ketoprofen, carprofen) in cattle on dairy farms. The question lists some procedures/conditions commonly dealt with in cattle that you **may or may not** consider require NSAIDs. Tick **one box per question**, for each procedure/condition.

**Procedure/ condition**	**Would you provide NSAIDs for the following conditions**	**If yes, for what proportion of cases would you use NSAIDs**	**What would you consider an ACCEPTABLE TOTAL cost for a course of analgesia for each procedure/ condition?**				
			**0€**	**€0 - €5**	**€5 - €15**	**€15 -€30**	**> €30**
Treatment of a sole ulcer (Cow)	Yes ☐ No ☐	%	☐	☐	☐	☐	☐
Sole haemorrhage/ bruising (Cow)	Yes ☐ No ☐	%	☐	☐	☐	☐	☐
White line disease with sub-sole abscess (Cow)	Yes ☐ No ☐	%	☐	☐	☐	☐	☐
White line disease NO sub-sole abscess (Cow)	Yes ☐ No ☐	%	☐	☐	☐	☐	☐
Claw amputation (Cow)	Yes ☐ No ☐	%	☐	☐	☐	☐	☐
Caesarean section (Cow)	Yes ☐ No ☐	%	☐	☐	☐	☐	☐
Dystocia - foetal-maternal disproportion requiring traction (Cow)	Yes ☐ No ☐	%	☐	☐	☐	☐	☐
Left displaced abomasum surgery (Cow)	Yes ☐ No ☐	%	☐	☐	☐	☐	☐
Mastitis - clots in milk only (cow)	Yes ☐ No ☐	%	☐	☐	☐	☐	☐
Disbudding (Calf)	Yes ☐ No ☐	%	☐	☐	☐	☐	☐
Surgical castration (Calf)	Yes ☐ No ☐	%	☐	☐	☐	☐	☐
Castration with Burdizzo (Calf)	Yes ☐ No ☐	%	☐	☐	☐	☐	☐

PART 4: PAIN ASSESSMENT

In your opinion, how painful do you think the following conditions and procedures are for **adult dairy cows and calves**? Assume **NO** analgesic/anaesthetic agents are provided. Circle **ONE** number from 1 (no pain) to 10 (worst pain imaginable).

**Condition**	***No Pain***									***Worst pain***
Left displaced abomasum (Cow)	1	2	3	4	5	6	7	8	9	10
Neck callouses *e.g*. caused by feed barrier (Cow)	1	2	3	4	5	6	7	8	9	10
Acute metritis (Cow)	1	2	3	4	5	6	7	8	9	10
Swollen hock (Cow)	1	2	3	4	5	6	7	8	9	10
Hock with hair loss (Cow)	1	2	3	4	5	6	7	8	9	10
Acute toxic *E-coli* mastitis (Cow)	1	2	3	4	5	6	7	8	9	10
Mastitis - clots in milk only (Cow)	1	2	3	4	5	6	7	8	9	10
Digital dermatitis (Cow)	1	2	3	4	5	6	7	8	9	10
White line disease with sub-sole abscess (Cow)	1	2	3	4	5	6	7	8	9	10
White line disease NO sub-sole abscess (Cow)	1	2	3	4	5	6	7	8	9	10
Sole haemorrhage/ bruising (Cow)	1	2	3	4	5	6	7	8	9	10
Calf pneumonia	1	2	3	4	5	6	7	8	9	10

**Procedure**	***No Pain***									***Worst pain***
Treatment of a sole ulcer (Cow)	1	2	3	4	5	6	7	8	9	10
Claw amputation (Cow)	1	2	3	4	5	6	7	8	9	10
Caesarean section (Cow)	1	2	3	4	5	6	7	8	9	10
Dystocia - foetal-maternal disproportion requiring traction (Cow)	1	2	3	4	5	6	7	8	9	10
Left displaced abomasum surgery (Cow)	1	2	3	4	5	6	7	8	9	10
Calving - no assistance required (Cow)	1	2	3	4	5	6	7	8	9	10
Disbudding (Calf)	1	2	3	4	5	6	7	8	9	10
Surgical castration (Calf)	1	2	3	4	5	6	7	8	9	10
Castration with Burdizzo (Calf)	1	2	3	4	5	6	7	8	9	10

PART 5: EMPATHY QUESTIONS

For each statement below, circle **ONE** number from 0 (does not describe me well) to 4 (describes me well).

**Statement (ANIMAL VERSION)**	***Does NOT describe me well***				***Describes me very well***
I often have tender, concerned feelings for animals less fortunate than others.	0	1	2	3	4
I sometimes find it difficult to see things from the animals point to view.	0	1	2	3	4
Sometimes I don't feel very sorry for animals when they have problems or suffer.	0	1	2	3	4
I try to understand the reasons behind an animal's undesired behaviour before making a decision.	0	1	2	3	4
When I see an animal being treated badly, I feel protective towards it.	0	1	2	3	4
I sometimes try to understand animals better by imagining how things look from their perspective.	0	1	2	3	4
Animals' misfortunes do not usually disturb me a great deal.	0	1	2	3	4
If I'm sure I'm right about how to handle an animal, I don't waste time trying to think what might be causing the animals behaviour.	0	1	2	3	4
When I see animals being treated unfairly, I sometimes don't feel very much pity for them.	0	1	2	3	4
I am often quite touched by things that I see happen.	0	1	2	3	4
I believe that there are two sides to every question and try to look at them both.	0	1	2	3	4
I would describe myself as an animal lover.	0	1	2	3	4
When I am disappointed or angry because of how an animal behaves, I usually try to put myself in its place for a while.	0	1	2	3	4
Before scolding an animal, I try to imagine how I would feel if I were in its place.	0	1	2	3	4

PART 6: LAMENESSS IN DAIRY COWS

Please circle the answer or fill in the grey box as required:

1. **How involved with lameness management have you been in the last year (circle <u>all</u> that apply)?**

   None Treating lame cows Putting preventative measures in place (i.e. footbathing)

   Identifying areas of risk for lameness on farms Lameness detection (i.e. lameness scoring)

   Creating lameness management plans

2. **Do you consider your knowledge in lameness management adequate?**

   Yes No Unsure

3. **Have you undertaken any CPD in pain management?**

   Yes No

4. **Have you undertaken any CPD in lameness?**

   Yes No

5. **Do you enjoy/ want to do lameness work on dairy farms?**

   Yes No

   If 'NO', why not?

6. **Where have you obtained most of your knowledge about lameness in dairy cows (please circle <u>one</u> only)?**

   Undergraduate training (vet school) Journals /articles Continuing education lectures

   Experience gained in practice Commercial literature / data sheets

   Other:

7. **Do you feel like there are enough opportunities to upskill in the area of dairy cow lameness?**

   Yes No

   If NO, what opportunities would you like available?

8. **Do you feel like farmers are doing enough to reduce lameness in their herd?**

   Yes No Unsure

9. **Do you think farmers would benefit from having a written herd health plan including lameness protocols (i.e. methods for detecting lameness, what to do if they detect a lame cow, methods of preventing lameness such as footbathing etc.)?**

   Yes No Unsure

10. What percentage of your dairy farmers have you discussed lameness with in the last year?

At herd level e.g. footbathing: %

At cow level e.g. treating individual cow: %

11. What do you think is the main reason that prevents farmers from doing more to reduce lameness in their herd?

12. What hoof lesion do you believe is the biggest cause of lameness on farms you have treated?

13. How would you treat a white line lesion?

14. How would you treat digital dermatitis?

Thank you for giving your time to complete this questionnaire

Please return using the pre-paid envelope
